# Heparan sulfate proteoglycan induces the production of NO and TNF-α by murine microglia

**DOI:** 10.1186/1742-4933-2-11

**Published:** 2005-07-16

**Authors:** Simona Bussini, Lucia Meda, Elio Scarpini, Emilio Clementi, Giancarlo Conti, Marco Tiriticco, Nereo Bresolin, Pierluigi Baron

**Affiliations:** 1 Department of Neurological Sciences, Centre for Excellence on Neurodegenerative Diseases and "Dino Ferrari" Center, University of Milan, Fondazione IRCCS "Ospedale Maggiore Policlinico, Mangiagalli e Regina Elena", Via F. Sforza 35, 20122 Milan, Italy; 2 Dept. Preclinical Sciences, University of Milano, 20157 - Milano and E.Medea Scientific Institute 23842 - Bosisio Pasini, Italy

## Abstract

**Background:**

A common feature of Alzheimer's disease (AD) pathology is the abundance of activated microglia in neuritic plaques containing amyloid-beta protein (Aβ) and associated molecules including heparan sulfate proteoglycan (HSPG). Besides the role as pathological chaperone favouring amyloidogenesis, little is known about whether or not HSPG can induce microglial activation. Cultures of primary murine microglia were used to assess the effect of HSPG on production of proinflammatory molecules that are known to be present in neuritic plaques of AD.

**Results:**

HSPG stimulated up-regulation of tumor necrosis factor-alpha (TNF-α), production of inducible nitric oxide synthase (iNOS) mRNA and accumulation of TNF-α protein and nitrite (NO_2_^-^) in a time- and concentration-dependent manner. The effects of HSPG were primarily due to the property of the protein core as indicated by the lack of microglial accumulation of TNF-α and NO_2_^- ^in response to denaturated HSPG or heparan sulfate GAG chains (HS).

**Conclusion:**

These data demonstrate that HSPG may contribute to chronic microglial activation and neurodegeneration seen in neuritic plaques of AD.

## Introduction

Senile plaques in the Alzheimer disease (AD) brain are characterized by the presence of an amyloid core consisting of fibrillar Aβ, surrounded by a wreath of dystrophic neurites and activated microglial cells. Reactive microglia release a variety of potentially neurotoxic compounds, including cytokines and free radicals. Many research groups have provided evidence that deposition of aggregated Aβ is centrally involved in the chronic inflammatory process occurring in senile plaques of AD brain [1,2].

Aβ is a 39- to 42-amino-acid peptide that arises from proteolytic processing of the amyloid precursor protein (APP) [3,4]. The Aβ peptide that is found in the senile plaques and cerebrovascular deposits exists as a multimeric aggregate with a fibrillar appearance [5]. Several other molecules have also been shown to be associated with Aβ deposits, and in vitro studies of fibrillogenesis suggest they may be important in the aggregation and persistence of the Aβ fibrils in vivo. These include apolipoprotein E, laminin, acetylcholinesterase, α_1_-antichymotrypsin and heparan sulfate proteoglycan (HSPG) [6-11].

HSPG is a multifunctional macromolecule characterized by a core polypeptide to which glycosaminoglycans (GAGs) are covalently attached. There are at least four different classes of HSPG present in AD, which are either associated with the cell membrane or with the extracellular matrix [12]. HSPG has been consistently associated with both diffuse and neuritic plaques [13,14]. Its early presence in AD pathological alterations as well as its immunohistochemical colocalization with all varieties of Aβ plaques, irrespective of their stage of maturation, have suggested that HSPG could play an active role in plaque formation. In this regard it has been proposed that HSPG facilitates Aβ deposition and/or promotes Aβ persistence by inhibiting clearance mechanisms, thus augmenting the formation of Aβ deposits in AD [15]. Consistent with this hypothesis, in vitro studies have shown that HSPG can bind with high affinity to Aβ as well as to APP and it protects Aβ from protease degradation [16-19].

Besides its function in amyloidogenic pathways, HSPG might contribute to AD pathogenesis also through activation of microglial cells. This possibility has never been investigated. To study this we have assessed in "in vitro" cultures of mouse microglial cells, two known markers of their activation, i.e. production of the proinflammatory cytokine tumor necrosis factor-α (TNF-α) and expression of the mRNA for the inducible nitric oxide (NO) synthase (iNOS). This enzyme is generated in microglia in response to a variety of pro-inflammatory cytokines and bacterial products, such as lipopolysaccharide (LPS). In addition, we have measured in the culture media supernatants the accumulation of NO_2_^-^, a good proxy for generation of NO by iNOS. Our results show that HSPG might contribute to neurodegeneration in neuritic plaques of AD also through activation of microglial cells and the ensuing increased inflammatory response.

## Materials and methods

### Reagents

Heparan sulfate proteoglycan (HSPG), heparan sulfate (HS), lipopolysaccharide (LPS, from Escherichia coli 026.B6) were purchased from Sigma (St Louis, MO, USA) and dissolved in clinical pyrogen-free H_2_O. Levels of endotoxin in HSPG and HS stocks were measured by E-TOXATE (Limulus Amebocyte Lysate, LAL) kit purchased from Sigma (St Louis, MO, USA).

### Preparation of Microglial Cultures

Mice were obtained from Charles River Laboratories, Inc. (Wilmington, MA, USA) and were used according to institutional guidelines that are in compliance with national (D.I. no. 116, G.U. suppl. 40, Feb. 18, 1992, Circolare No.8, G.U., 14 Luglio 1994) and international law and policies (EEC Council Directive 86/609, OJ L358, 1 Dec. 12, 1987; Guide for the Care and Use of Laboratory Animals, U.S. National Research Council, 1996). Primary murine microglial cultures were prepared as previously described [20]. Briefly, cerebral cortical cells from day 1-old mice were dissociated with 0.25% trypsin and 0.1% DNAse (Sigma) and plated in 75 cm^2 ^culture flasks (Corning, Acton, MA) in Dulbecco's Modified Eagle's Medium (DMEM) (Invitrogen Corporation Grand Island, NY, USA) containing 10% heat-inactivated FBS (Invitrogen Corporation Grand Island, NY, USA) and 100 μg/ml gentamicin (Invitrogen Corporation Grand Island, NY, USA). Dissociated glial cultures were maintained at 37°C with 5% CO_2 _and medium was replenished 4 days after plating. On day twelve of culture, flasks were shaken for 2 hours. The culture media supernatants (containing predominantly microglia and oligodendrocytes) were then collected and the cells were plated in either 96-well tissue plates (Nunc, Roskild, DK) at a concentration of 4 × 10^4 ^cells per 100 μl per well, or in 48-well tissue plates at a concentration of 25 × 10^4 ^per 500 μl per well, and maintained at 37°C with 5% CO_2 _for 1 hour. Loosely adherent oligodendrocytes were then removed from the cultures by gentle shaking of the culture plates by hand. After pouring off the culture media supernatants (containing oligodendrocytes), the adherent microglia were maintained at 37°C with 5% CO_2 _for subsequent treatment. The purity of microglial cultures was routinely assessed by staining with the F4/80-antibody (Serotec, Oxford, UK), which recognizes a glycoprotein expressed predominantly in microglia/macrophages cells [21], and found to be in the range of 96–98% in all cell preparations.

### Exposure of microglia to HSPG

Microglial cells were incubated with HSPG at a concentration of 5, 15, 30, 40 μg/ml for 2, 4, 12, 24 and 48 h. After the indicated times, culture supernatants were harvested, and frozen at -70°C until assayed for levels of secreted TNF-α and nitrite (NO_2_^-^). After 4 h total RNA for each conditions was isolated from cells plated in 48-well tissue culture plates, using the TRIzol reagent protocol (Invitrogen; Carlsbad, CA, USA). In some experiments cells were exposed to 15 μg/ml of HS or HSPG denaturated at 90°C for 10 min. After treatment at indicated times, culture media supernatants were assayed for NO_2_^- ^accumulation and TNF-α release as described below.

### TNF-α assay

Antigenic mouse TNF-α was detected by ELISA system from Biosurce (Camarillo, CA, USA), and based on the quantitative "sandwich" enzyme immunoassay technique. The sensitivity of the assay was 10 pg/ml.

### Nitrite assay

Nitrite (NO_2_^-^) is a stable end-product used extensively as an indicator of NO production by cultured cells. In our experimental conditions, NO_2_^- ^accumulation was assayed by the Griess reaction, according to the method previously described [22]. Briefly, culture media supernatants were mixed with equal amounts of Griess reagent (p-aminobenzene sulfonamide 1%, naphtylethylenediamide 0.1% in phosphoric acid 2.5%) in 96-well plates: samples were incubated at room temperature for 10 min, and subsequently absorbance was read at 540 nm using a microplate reader. NO_2_^- ^concentrations were calculated in accordance with a sodium nitrite standard curve.

### RT-PCR

One μg total RNA quantitated spectrophotometrically and isolated from each condition was reverse transcribed using oligo(dT)_20_-primers and Superscript II-Reverse Transcriptase according to the manifacturer's protocol (Invitrogen, Carlsbad, CA, USA). c-DNA equivalent to 20 ng of total RNA was subjected to subsequent PCR analysis in a total volume of 30 μl containing 25 pmol of primers specific for TNF-α, iNOS and glyceraldehyde phosphate dehydrogenase (GAPDH; used as an internal control) (Table I), 10 mM Tris-HCl pH 8.3 (at 25°C), 50 mM KCl, 10% DMSO, 1.25 mM MgCl_2_, 250 μM each of dATP, dCTP, dGTP, dTTP, and 1.5 units AmpliTaq DNA-polymerase (Roche, Branchburg, NJ, USA). PCR was performed at the following conditions: (1) 2 min at 93°C; (2) 30 sec at 93°C, 30 sec at 60°C (TNF-α) or at 68°C (iNOS and GAPDH), 45 sec at 72°C for 26 cycles (iNOS), 24 cycles (TNF-α) or 20 cycles (GAPDH); (3) 10 min. at 72°C. PCR products were analysed on 1.5% agarose gel containing 10 μg/ml ethidium bromide. Controls included RNA subjected to the RT-PCR procedure without addition of reverse transcriptase and PCR performed in the absence of c-DNA which always yelded negative results.

**Table I:** Oligonucleotide Primers Used for cDNA amplification

Probe	Cycles	Orientation	Tann	Sequence	Length of PCR Fragments (bp)
GAPDH	20	sense antisense	68	5'TGAAGGTCGGTGTGAACGGATTTGGC3' 5'CATGTAGGCCATGAGGTCCACCAC3'	983
iNOS	26	sense antisense	68	5'CCCTTCCGAAGTTTCTGGCAGCAGC3' 5'GGCTGTCAGAGCCTCGTGGCTTTGG3'	493
TNFα	24	sense antisense	60	5'TTCTGTCTACTGAACTTCGGGTGATCGGTCC3' 5'GTATGAGATAGCAAATCGGCTGACGGTGTGGG3'	378

### Statistical analysis

Data are expressed as means ± standard deviations (SD). Statistical evaluation was performed by repeated measures ANOVA (analysis of variance) followed by Dunnet's test for specific comparisons. Statistical significance was set at *P *< 0.05.

## Results

### 1. HSPG triggers production of NO_2_^- ^and TNF-α in cultured microglia

**Figure 1:**
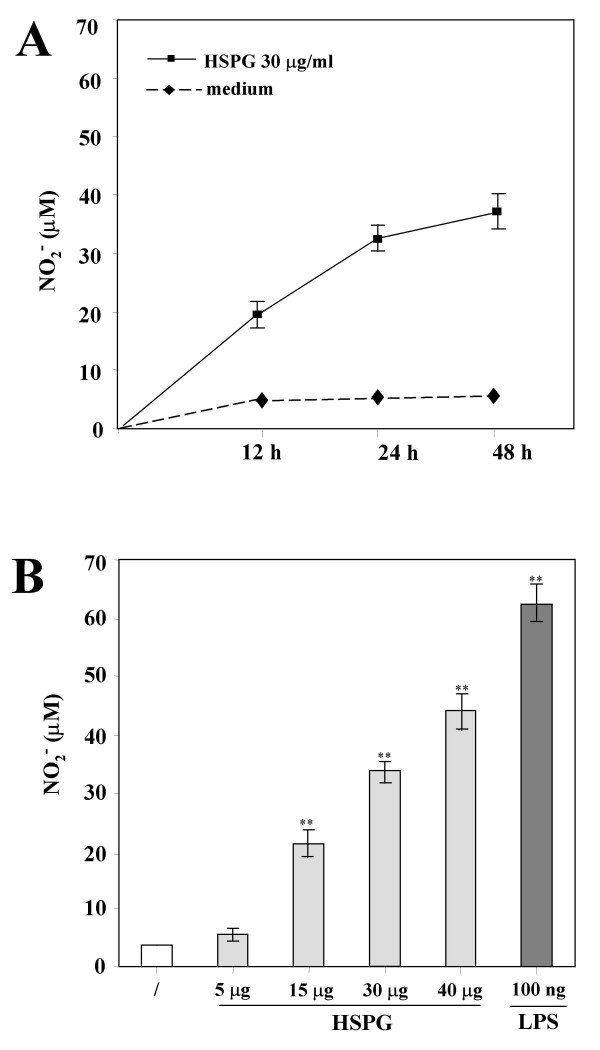
**Effect of HSPG on the accumulation of NO_2_^- ^from murine microglia. **In **panel A**, time course of NO_2_^- ^production by murine microglia in response to HSPG. Microglial cells were cultured in 96-well plates and stimulated with 30 μg/ml HSPG for up to 48 h. In **panel B**, dose-dependent effect of HSPG on the accumulation of NO_2_^- ^by murine microglia. Microglial cells were cultured in 96-well plates and stimulated for 24 h with increasing concentrations of HSPG, or 100 ng/ml LPS. Mean values ± SD of assays performed with culture media supernatants collected and pooled from triplicate wells for each condition are shown (n = 9). Both panels depict a representative experiment out of three performed with similar results. **p < 0.01.

**Figure 2:**
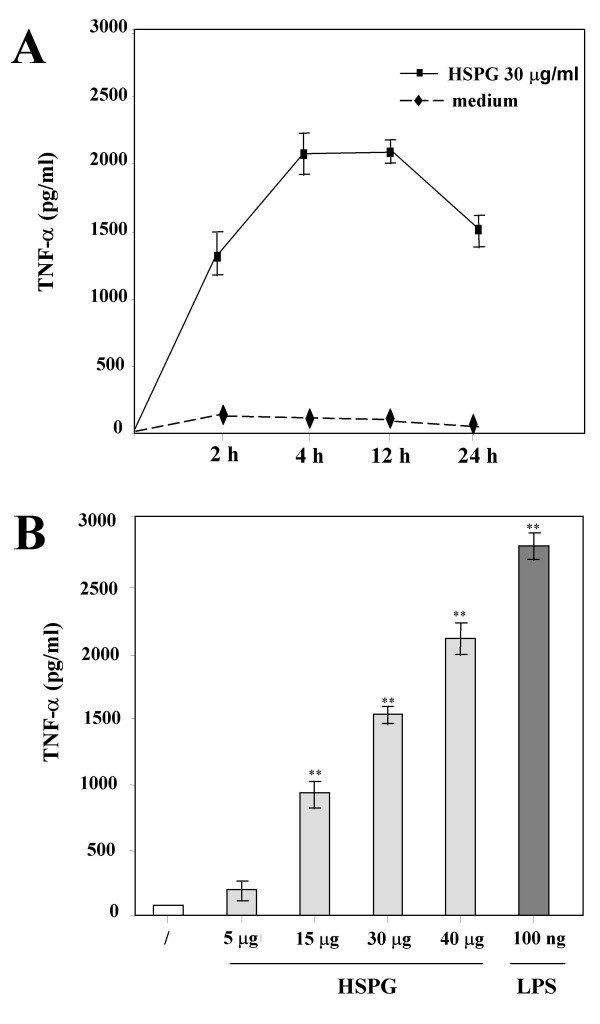
**Effect of HSPG on TNF-α release by murine microglia. **In **panel A**, time course of TNF-α production by murine microglia in response to HSPG. Microglial cells were cultured in 96-well plates and stimulated with 30 μg/ml HSPG for up to 24 h. In **panel B**, dose-dependent effect of HSPG on the accumulation of TNF-α by murine microglia. Microglial cells were cultured in 96-well plates and stimulated for 24 h with increasing concentrations of HSPG or 100 ng/ml LPS. Mean values ± SD of assays performed with culture media supernatants collected and pooled from triplicate wells for each condition are shown (n = 9). Both panels depict a representative experiment out of three performed with similar results. **p < 0.01.

To test whether the interaction of HSPG with microglia could induce the production of proinflammatory and potentially cytotoxic mediators, we assayed the accumulation of NO_2_^- ^as an indirect measure of NO production from mouse primary microglia stimulated with HSPG and, for comparison, with LPS. As shown in Fig. 1, microglia in resting conditions did not release detectable NO_2_^- ^even after a 48-h incubation, whereas HSPG induced significant accumulation of NO_2_^- ^in culture media supernatants in the range of that observed with LPS. The effect of HSPG on NO_2_^- ^production was time and concentration-dependent, with maximal accumulation observed at 48 h (7.5. ± 0.5-fold increase over control at 30 μg/ml HSPG; P < 0.05, n = 9) (Fig. 1A) and production already significantly increased after a 24 h exposure to 15 μg/ml (5.2 ± 0.4-fold increase over control; P < 0.05, n = 9) (Fig. 1B). We also investigated whether or not HSPG was able to induce the production of TNF-α by microglia. Untreated cells constitutively produced very small amounts of TNF-α, whereas their stimulation with HSPG or LPS resulted in the release of significant levels of TNF-α, with maximal accumulation observed at 4 h (25 ± 1.8-fold increase over control; P < 0.05, n = 9), followed by a decrease at later times (Fig. 2A). Concentration-response studies demonstrated that the amount of TNF-α released into the culture media supernatants increased with increasing concentrations of HSPG (Fig. 2B). TNF-α release was already significantly increased after a 24 h exposure to 15 μg/ml HSPG (11.6 ± 0.85.-fold increase over control; P < 0.05, n = 9). Specificity of the effects of HSPG on microglial activation was confirmed by LAL test that excluded trace levels of endotoxin in our HSPG stocks.

### 2. HSPG induces expression of iNOS and TNF-α mRNA in cultured microglia

**Figure 3:**
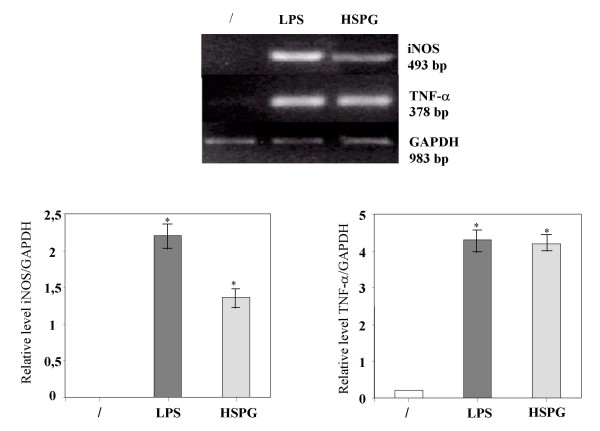
**Effect of HSPG on iNOS and TNF-α mRNA expression in murine microglia. **One-day-old microglial cells were cultured in 48-well plates and then stimulated for 4 hours with 15 μg/ml HSPG or 100 ng/ml LPS. Total RNA was extracted and analysed by RT-PCR. The densitometric evaluation of the bands obtained from three independent experiments in duplicates are reported (n = 6); values are expressed as the relative level of iNOS/GAPDH and TNFα/GAPDH. *p < 0.05.

To determine whether the production of NO_2_^- ^and TNF-α triggered by HSPG reflected induction of iNOS and TNF-α mRNA, RT-PCR analysis was performed on microglia total RNA, using probes complementary to the mouse macrophage iNOS and TNF-α coding sequences. In resting conditions the mRNA expression for iNOS and TNF-α in microglia was absent. On the contrary, mRNA levels for iNOS and TNF-α were clearly induced after stimulation of the cells for 4 h with HSPG at the concentration of 15 μg/ml or 100 ng/ml LPS (Fig. 3).

### 3. Microglial activation induced by HSPG is mediated primarily by the protein core

**Figure 4:**
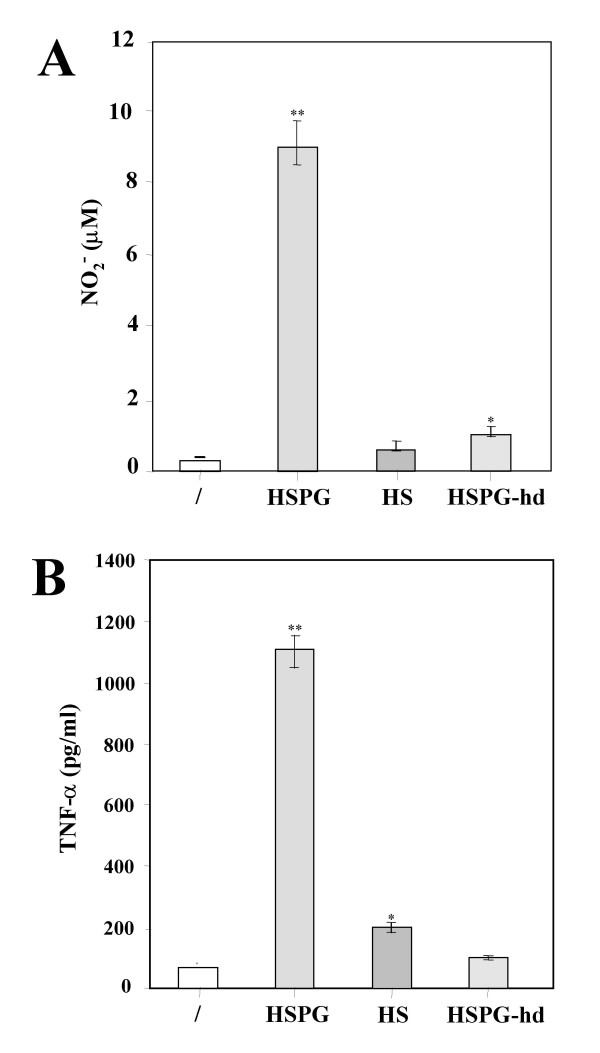
**Effect of heat denaturated HSPG and HS-GAG chains on NO_2_^- ^and TNF-α release by murine microglia. **Microglial cells were cultured in 96-well plates and stimulated with 15 μg/ml HSPG, 15 μg/ml heat denaturated HSPG (HSPG-hd) or 15 μg/ml HS-GAG chains (HS). After 24 h, culture media supernatants were assayed for NO_2_^- ^accumulation (**A**) and TNF-α release (**B**). Mean values ± SD of assays performed with culture media supernatants collected and pooled from triplicate wells for each condition are shown (n = 9). Both panels depict a representative experiment out of three performed with similar results. *p < 0.05, **p < 0.01.

**Figure 5:**
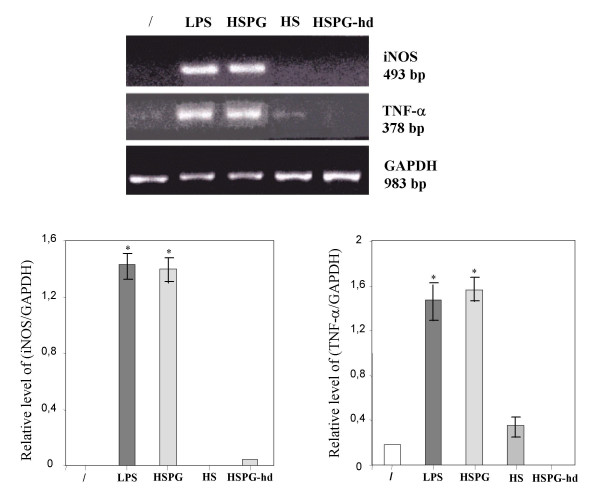
**Effect of heat denaturated HSPG and HS-GAG chains on iNOS and TNF-α mRNA expression in murine microglia. **Microglial cells were cultured in 48-well plates and then stimulated for 4 hours with 15 μg/ml HSPG, 15 μg/ml heat denaturated HSPG (HSPG-hd), 15 μg/ml HS-GAG chains (HS) or 100 ng/ml LPS. Total RNA was extracted and analysed by RT-PCR. The densitometric evaluation of the bands obtained from three independent experiments in duplicates are reported (n = 6); values are expressed as the relative level of iNOS/GAPDH and TNF-α/GAPDH. *p < 0.05.

To examine whether the protein core or the GAGs of HSPG were involved in mediating microglial activation, the effects of HSPG were compared with those obtained using HSPG denaturated at 90°C for 10 min (HSPG-hd) or HS-GAG chains (HS). As shown in Fig. 4, exposure of microglia to 30 μg/ml HSPG-hd almost completely abolished the production of NO_2_^- ^(1 ± 0.2 μM; P < 0.05, n = 9) and particularly of TNF-α (98 ± 10 pg/ml; n = 9). Stimulation with 30 μg/ml HS only slightly affected release of TNF-α (200 ± 18 pg/ml; P < 0.05, n = 9) but not accumulation of NO_2_^- ^(0.8 ± 0.1 μM; n = 9). The lack of ability of HSPG-hd and HS to maintain NO_2_^- ^accumulation and TNF-α release appeared to be mediated at the transcriptional level since HSPG-hd and HS failed to increase both iNOS and TNF-α mRNA transcription (Fig. 5).

## Discussion

The identification in senile plaques of pathologic stimuli that can lead to microglial activation represents one of the important issues of immunologic research in AD. Previous studies have shown that microglia upon stimulation with Aβ can produce proinflammatory and cytotoxic mediators, and that these mediators play a role in the pathogenesis of AD [23-26]. The demonstration of the indirect neuronal injury prompted us to investigate if, in addition to Aβ, others molecules present in senile plaques could be involved in similar mechanisms of microglial activation. We have identified HSPG as a potential candidate in this process on the basis that this molecule has been found to be associated with Aβ peptide-containing deposits and suggested to act as pathological chaperone, increasing β-pleated structure within Aβ [27,28].

We demonstrate that HSPG is able to induce the release of NO_2_^- ^and TNF-α by cultured primary murine microglia. By assessing iNOS and TNF-α mRNA expression with RT-PCR we have also shown that the release of NO and TNF-α in HSPG-stimulated microglial cells is due to the induction of iNOS and TNF-α gene expression. These findings suggest that, in addition to Aβ, also HSPG is able to activate microglia with production of proinflammatory molecules known to be present in the brain of AD patients.

The precise mechanism by which microglia mediate neuronal cell injury in AD is incompletely understood and several mediators have been proposed, among them NO and TNF-α [29-31]. Even though physiological levels of NO may influence synaptic efficacy by regulating neurotrasmitter release [32], excess NO may cause neuronal degeneration by combining with oxygen radicals such as superoxide anion to form the highly toxic peroxynitrite ion [33]. Similarly, TNF-α has been reported to be trophic to rat hippocampal neurons [34]. However, transgenic mice that overexpress TNF-α exibit severe inflammation and neurodegeneration [35]. Moreover, in vitro studies have shown that some Aβ-induced microglial activities, including neurotoxicity and chemokine production, are mediated through release of endogenous TNF-α [23,36,37]. That TNF-α is presumably involved in AD pathology is also supported by its elevated levels in the serum, CSF and cerebral cortex of AD patients [38,39]. In view of the information summarized above, it is conceivable that generation of NO and TNF-α from microglial cells is one means by which HSPG may enhance the inflammatory reaction in neuritic plaques, and thus pathogenesis of AD. However, it must be pointed out that astrocytes represent the main source of NO in the plaques whereas release of neurotoxic levels of NO by microglia has been shown in vitro [40,23,24,41]. Future studies will be needed in order to determine whether HSPG also activates astrocytes to produce NO and TNF-α.

The lack of inflammation and microglial activation described in diffuse plaques opens the question about the actual proinflammatory role played by HSPG at early stages of plaque evolution. Our results show that activation of microglial cells by HSPG is concentration-dependent, both in terms of NO_2_^- ^accumulation and TNF-α release in the culture medium, and that 15 μg/ml of the compound is sufficient to trigger the activation process. Although HSPG has been immunohistologically localized to senile plaques its biochemical isolation from these structures, and thus its quantification, have yet to be performed [42]. Thus, we cannot establish at present whether the threshold for microglial activation we found can explain the discrepancies in immunohistochemical localization of HSPG in diffuse plaques lacking inflammation.

The cellular source of HSPG in mammalian brain is represented by microglia and astrocytes which have been shown by immunofluorescence and/or western blotting to express HSPG both in vitro and in vivo [43,44]. Nevertheless, the factors implicated in HSPG biosynthesis and deposition have been poorly investigated. Recently regulation of HSPG by injury and IL-1α has been demonstrated in astrocytes and microglia [45]. Therefore, the stimulation of microglial cells by HSPG, followed by increased production of proinflammatory cytokines could stimulate further HSPG formation in an autocrine, feed-forward manner.

The importance of HSPG deposition in AD pathogenesis is supported by the fact that HSPG binds Aβ, accelerates Aβ fibril formation and maintains Aβ fibril stability [46]. Most of these effects of HSPG have been shown to be due to its associated HSGAG side-chains, as also suggested by the lack of extracellular Aβ deposits in transgenic mice overexpressing HSPG protein core [47]. This contrasts with our results showing that the proinflammatory role of HSPG is primarily mediated by its protein core. However, in these transgenic mice HSPG was detected only inside the cells, i.e. without accumulation of the compound in the extracellular environment, which instead is a hallmark of AD neurodegeneration. Studies in these animals, therefore, do not allow drawing conclusions about the role of extracellular HSPG in AD.

In conclusion, the potential participation of HSPG in NO and TNF-α-mediated neuronal injury induced by microglia adds a novel biological role of this molecule in the pathogenesis of AD. These data indicate that HSPG plays an immunomodulatory role in the activation of microglia, in addition to that proposed in amyloidogenic pathways and demonstrate another mechanism by which immune responses may be triggered in AD brain. Therefore, a further understanding of the role of HSPG in the pathogenesis of AD would assist in the development of rational, targeted therapeutic strategies to combat this neurodegenerative disorder.
